# The Machinability of Different Albromet W130 Plates Thicknesses by WEDM to the Required Surface Roughness Value

**DOI:** 10.3390/ma17225520

**Published:** 2024-11-12

**Authors:** Katerina Mouralova, Libor Benes, Radim Zahradnicek, Jiří Fries, Andrea Manova

**Affiliations:** 1 Faculty of Mechanical Engineering, Brno University of Technology, 602 00 Brno, Czech Republic; zahradnicek@vutbr.cz; 2 Faculty of Mechanical Engineering, Jan Evangelista Purkyně University, 400 96 Ústí nad Labem, Czech Republic; libor.benes@ujep.cz (L.B.); amanova@seznam.cz (A.M.); 3 Department of Machine and Industrial Design, VSB—Technical University of Ostrava, 708 00 Ostrava, Czech Republic; jiri.fries@vsb.cz

**Keywords:** WEDM, wire electrical discharge machining, thicknesses, Albromet W130, design of experiment, surface roughness

## Abstract

Wire Electrical Discharge Machining (WEDM) technology represents an unconventional but vital manufacturing technology in many different industrial branches. The automotive industry and its many significant requirements bring the need to manufacture inserts and mould segments for plastic injections from Albromet W130 material, with a required roughness, Ra, from 4.5 to 5 µm so that subsequent profile etching can be eliminated. A planned experiment of 60 rounds was carried out to discover the optimal machining parameters, namely, the pulse-off time, gap voltage, discharge current, pulse-on time, and wire speed in order for the thickness of 10 to 100 mm (after 10 mm) to demonstrate the required roughness. The goal was to evaluate the surface roughness, maximise the cutting speed, and manufacture it without surface or subsurface defects. The evaluation of the planned experiment led to the establishment of optimised WEDM machining parameters with which thicknesses of 10–100 mm will always be produced with the required roughness, Ra, from 4.5 to 5 µm and with the highest possible cutting speed. It was also proven that the machining does not lead to surface or subsurface defects, and thus, the service life of the manufactured parts will not be affected.

## 1. Introduction

Wire Electrical Discharge Machining (WEDM) is an unconventional machining technology that is widely used in aviation, automotive, military, and medical industries [1]. WEDM is based on the principle of material removal using physical laws. The only requirement is at least a minimal electrical conductivity of the machined material. It is thus possible to machine materials that are difficult to machine conventionally, e.g., due to their high hardness (e.g., after heat treatment), high toughness, or thin and soft profiles [2]. The machining principle lies in the use of a tool, which is a thin wire with a diameter of 0.3 to 0.02 mm, which never physically touches the workpiece. There is always a so-called spark gap left between them, which contains the dielectric fluid, a liquid with high electrical resistance (most often deionised water) [3]. The material removal is relatively slow (1–20 mm/min) and depends mainly on the thickness of the material and its type. That means that WEDM is a relatively energy-consuming machining technology, and so its optimisation becomes essential. In order to reach this optimisation, planned experiments are carried out to increase the cutting speed, thereby reducing the machining time while meeting the requirements for the machined surface [4].

Copper and copper alloys are some of the most versatile engineering materials available. A combination of physical properties, such as strength, conductivity, corrosion resistance, machinability, and ductility, make copper suitable for a wide range of applications. These properties can be further improved by variations in composition and manufacturing methods [5].

Sathiyaraj [6] examined the WEDM performance on copper alloys. For the performance to be assessed, the material removal rate (MRR) and surface roughness (Ra) were used as indicators. The experiment was led using Taguchi’s L9 orthogonal array and different combinations of three machining parameters, e.g., the pulse-on time, pulse-off time, and peak current. Their combinations were analysed with a signal-to-noise (S/N) ratio, analysis of means, and analysis of variance (ANOVA). Results have shown that the peak current and pulse-on time significantly impact the MRR and surface roughness. Thankachan [7] investigated the characteristics of a friction-stir-processed (FSPed) copper-Boron Nitride (BN) composite machined using WEDM. The Taguchi Method and Grey Relational Analysis were employed. The outcomes from different machining parameters, including the pulse-on time, pulse-off time, and wire feed rate, and their responses to the MRR and Ra were studied. The ANOVA proved that the pulse-on time and BN volume fraction had the most influence on the MRR, and the pulse-on and -off time led to minimal Ra values. These findings enabled them to develop a reliable mathematical model to predict the responses. Katiyar [8] studied the synthesising of an iron–copper alloy by WEDM. The alloy is extremely difficult to synthesise; hence, there was an attempt to produce it through high temperature and subsequent rapid cooling that occurred during WEDM. X-ray diffraction analysis, scanning electron microscopy (SEM), energy dispersive spectroscopy (EDS), and transmission electron microscopy (TEM) were employed to analyse the generated debris. Various machining inputs were considered, and it was established that the pulse-on time impacts the shape of the particle the most, where a high pulse-on time will lead to a spherical shape. Spherical-shaped particles show a high amount of Fe, while non-spherical ones show high Cu contents. The particle size ranged between 50 nm and 30 µm. Li [9] researched the machining of a copper tungsten alloy by WEDM. They examined the influence of the following machining parameters: the pulse width, peak current, and servo voltage on the MRR and established an optimal combination, leading to the maximum MRR. The orthogonal test, range analysis, and variance analysis were used for the test data. The conclusion was that the peak current was the most significant parameter for the MRR. A maximum MRR of 25.28 mm^3^/min was obtained with the parameters’ combination: Ton 50 μs, IP 12A, and SV 8V. Li [10] studied the influence of various electrical parameters on the cutting speed and Ra of pure copper machined with WEDM. Ahmed [11] investigated the WEDM fabrication of large surface-area micro-channels from copper. Ali [12] studied the impact of WEDM parameters on the MRR of Beryllium Copper (BeCu). Bhuiyan [13] investigated the possibility of fabricating a copper-based electrostatic comb-drive actuator using WEDM with a low actuation voltage.

The relatively high energy demand of WEDM technology makes it vital to optimise the process of reducing machining times while maintaining the required quality of the machined surface, hence preserving the competitiveness of this unconventional machining technology. The aim of the study was to carefully analyse the different thicknesses of the Albromet W130 material machined by WEDM, which plays a key role in the cutting speed. The present extensive study was conducted to determine the machinability of different thicknesses of Albromet W130 material. The novelty of the study mainly lies in taking into account different thicknesses of the processed material, which fundamentally affect the cutting speed of the Albromet W130 copper alloy, as well as maintaining the required quality, which has not been published before. This study builds on and benefits from the knowledge gained from machining aluminium alloy 7475-T7351 [14], high entropy alloys [15], and a Nimonic C 263 nickel alloy [16]. The findings gained and presented in the study can be directly used in practice, such as when machining inserts and segments of plastic injection moulds, which are indispensable in the automotive industry.

## 2. Materials and Methods

### 2.1. Experimental Material

**Figure 1:**
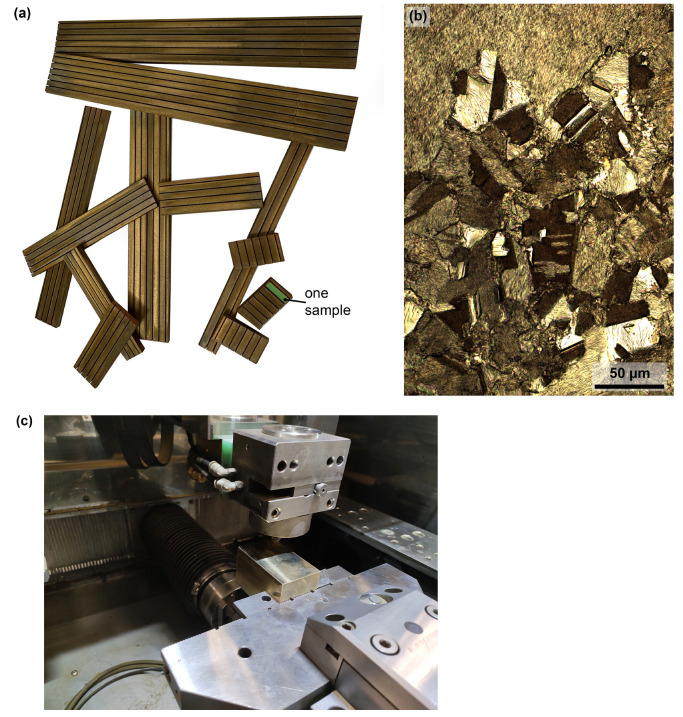
(**a**) Example of samples fabricated within the experiment, (**b**) microstructure of Albromet W130 (LM), (**c**) example of cutting in machine.

The experiment samples for this study are shown in Figure 1a. They were made of an Albromet W130 copper alloy with a chemical composition of 2% Be and a Cu balance by weight. This material was purchased from a regular dealer and was supplied with a material sheet. Figure 1b shows the microstructure displayed using light microscopy (LM). Section 2.2 describes the preparation of the metallographic preparations more closely. Albromet W130 is a heat-treated copper–beryllium alloy that has a thermal conductivity of 20 °C 130 W/m·k and a tensile strength of R_m_ = 1250 N/mm^2^. It has a high hardness of 340–390 HB 30 and a yield strength of R_p0.2_ = 1000 N/mm^2^. It is used to produce parts of moulds for plastic injection and electrodes intended for welding. Initial semi-finished product prisms with thicknesses of 10, 40, 50, 70, 90, 100, and 130 mm were used in the experiment, with the cut length of each sample always being 3 mm (shown in Figure 1a).

### 2.2. WEDM Machine Setup and Design of Experiment

A 5-axis Robocut C400iB wire-cutting machine from Fanuc (Oshino-mura, Yamanashi Prefecture, Japan) was used for the machining. This machine allows the workpiece to be immersed in deionised water throughout the entire machining process. The tool electrode was a brass wire type CUT P of 0.25 mm diameter, made by PENTA TRADING (Prague, Czech Republic).

The goal of this design of experiment (DoE) was to find such machining parameter settings for the desired value of surface roughness, Ra, in class 33, according to VDI standard 3402Blatt4: application of Electrical Discharge Machining would be achieved. The studied parameters were the pulse-off time (*T_off_*), gap voltage (*U*), discharge current (*I*), pulse-on time (*T_on_*), and wire speed (*v*). The norm required the Ra range to be from 4.5 to 5 µm for individual thicknesses (*t*) of the material. The design of the experiment was processed using the Stat-Ease 360 program by the American company Stat-Ease.

Quadratic factor effects were identified based on historical data from a previous DoE experiment, totalling 54 rounds. The resulting Ra values for all samples’ thicknesses ranged from approximately 2.5 to 3.5 µm. The highest Ra values of roughly 4.7 µm (considering all the measurement positions) were reached for the sample fabricated with the following machining parameters: *T_on_* = 8 µs, *T_off_* = 40 µs, *I* = 35 A, *U* = 50 V, *v* = 12 m/min, and sample thickness *t* = 130 mm. This sample was the only one that met the requirement for class 33, according to the norm.

**Figure 2:**
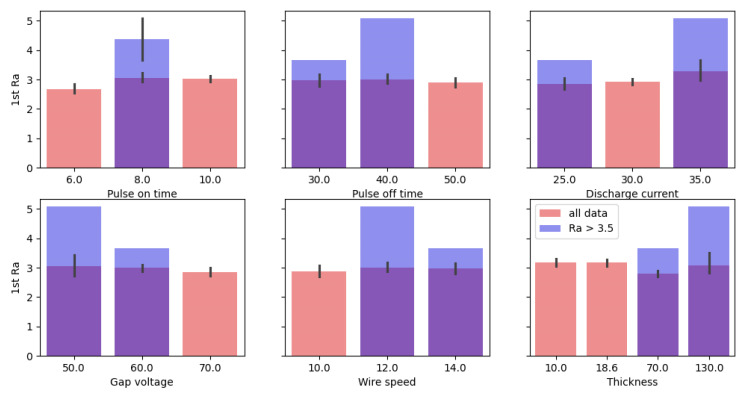
Comparison of the only two settings with Ra > 3.5 µm with the average Ra from the other runs.

A mathematical model for the Ra was established, where the most important effects were the quadratic effect of sample thickness, Thickness^2^, the linear effect of the discharge current, and the double interaction between the discharge current and gap voltage. We gained important information that the wire speed was conversely not a statistically significant factor in any of the models. Furthermore, an exploratory analysis of the data shown in Figure 2 indicated that higher Ra values could be expected at a pulse-on time of 8 µs, at lower gap voltage values, at higher thickness values, and at medium-to-higher wire speed settings.

**Figure 3:**
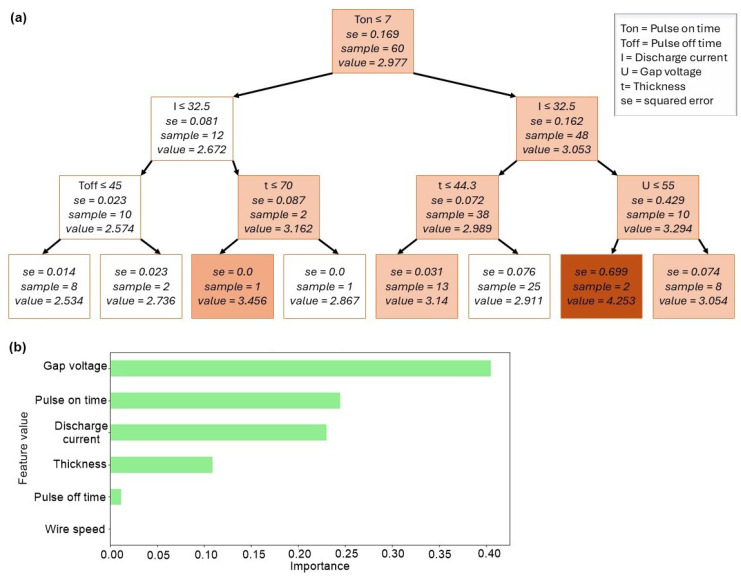
(**a**) Decision prescription of DecisionTreeRegressor algorithm with depth 3, (**b**) importance weight of effect on Ra response.

The DecisionTreeRegressor algorithm was applied to the historical data. Its principle is that, at each step, it divides the dataset into two parts according to the information benefit the division brings. At the end of this branching, the so-called leaves of the tree form, containing similar values with low entropy. The advantage of this algorithm is its simplicity and understandable output, as shown in Figure 3a, which is to be followed to achieve the result. From the prescription from the decision tree with a depth of 3 and the achieved coefficient of determination R^2^ = 0.56, the algorithm preferred the following settings: pulse-on time > 7 µs, discharge current > 32.5 A, and gap voltage <= 55 V. The graph in Figure 3b shows the importance of individual factors influencing the response, which makes it an important constituent of the algorithm. The algorithm assigns the greatest weight to the gap voltage factor and no weight to the wire speed factor, as can be seen in the graph. Once the above findings from the exploratory analysis were considered, it was decided that the unimportant factor of wire speed would not be included in the DoE’s continuation. Its setting throughout the experiment remained at the centre value of 12 m/min, which allowed for the best result. This step reduced the number of necessary design trials and enabled the addition of other trials, e.g., more precise repeated measurements (replicate points) and trials filling the experimental space (lack of fit points).

The DoE plan type ranks among the I-optimal designs, which are algorithmically designed for response surface methodology (RSM), where the prediction of the model is more important than the coefficients of individual parameters. The ‘I’ is derived from the word integrated because the algorithm selects plan points based on minimising the integrity of the variance of the prediction over the experimental space. These plans are called optimal because they are flexible, and their design can be adapted to the case at hand, which does not need to conform and adapt to more familiar DoE templates. Moreover, they are generated to preserve as much orthogonality as possible, meaning they preserve the independence of individual factor effects. The subsequent cubic model required 23 trials, and its design included 18 additional trials to the model (improving the orthogonality of the design), 10 trials filling the experimental space and checking the lack of fit (how well the model fits), 5 repeated replication trials, and 4 trials for the centre point of the plan for error estimation. There were 60 rounds planned for the design of the experiment. This design of the experiment contained 7 levels of sample thickness and is shown in Table 1. There were 5 investigated factors in total, as shown in Table 2. The setting limit values were obtained through extensive previous testing as well as from the machine manufacturer’s recommendations. Only a small number of wire electrode breaks occurred, namely for samples number 3, 5, 11, 13, 16, 24, 27, 31, 40, 46, 48, 50, 53, and 57. This problem was not addressed, as the WEDM machine disposes of a very fast automatic wire rethreading system.

### 2.3. Experimental Methods

All the samples produced for this experiment were cleaned in an ultrasonic cleaner and analysed with a scanning electron microscope LYRA3 by Tescan (Brno, Czech Republic). For the analysis of surface and subsurface microstructural changes and the measurement of the cut gap, metallographic preparations of the cross-sections were prepared from the WEDM machined material. They were prepared using common techniques—wet grinding and polishing with diamond pastes. The automatic preparational system TEGRAMIN 30 by Struers (Westlake, Cleveland, OH, USA) was employed. The final mechanical–chemical polishing was carried out using OP-Chem suspension from Struers. After being etched for 15 s with an etchant consisting of NH_4_OH + H_2_O + H_2_O_2_ in a 1:1:1 ratio, the structure of the material was observed and documented using electron and light microscopy of the Axio Observer Z1m inverted light microscope by ZEISS (Jena, Germany). The topography was studied with the Dektak XT 3D tactile profilometer by Bruker (Billerica, MA, USA). The measured data were later processed in Vision 64 software.

## 3. Results and Discussion

### 3.1. Cutting Speed Evaluation

**Figure 4:**
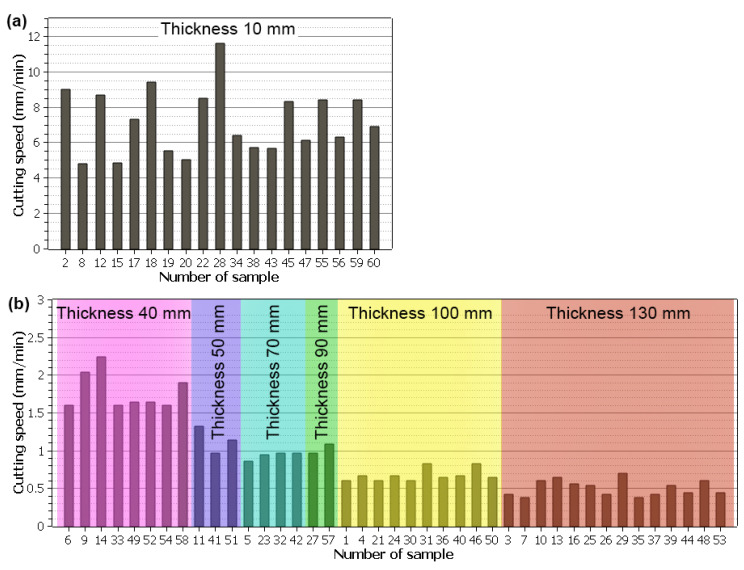
Cutting speeds of individual samples of different thicknesses, (**a**) sample thickness of 10 mm, (**b**) remaining thicknesses.

The cutting speed is usually set in a program in conventional machining machines. The WEDM machine, however, regulates the cutting speed itself by setting the parameters in such a way that the wire electrode and the workpiece would not come into physical contact, as that would lead to a short circuit. That is why setting and obtaining the highest possible cutting speed is the subject of WEDM process optimisation. The cutting speed depends not only on the machined material but also on the thickness of the workpiece. The cutting speed was read from the machine’s display throughout the cutting of each sample and thoroughly recorded. It was then compiled into graphs shown in Figure 4. Due to the significant change in the cutting speed for samples with the smallest thickness of 10 mm, these were plotted separately because of the Y-axis range. The cutting speed was significantly lower at higher thicknesses and thus would not be recognisable in the graph. The graphs show that with an increase in thickness, there is a corresponding decrease in the cutting speed. This fact is well-known from studies such as Mouralova [17] and Liao [18]. The highest cutting speed of 11.6 mm/min was seen in sample No. 28 with the following machining parameters: *T_on_* = 10 µs, *T_off_* = 50 µs, *I* = 25 A, *U* = 70 V, and *v* = 12 m/min. This cutting speed is relatively high compared to studies by Manoj [19] and Singh [20], where different materials were machined at an equal thickness of 10 mm. A different workpiece material can be the reason for this, as well as the fact that the WEDM machines in those studies have older generators that are not as powerful.

For the cutting speed(*v_c_*) response, the backward selection was used to improve the criteria of BIC (Bayesian information criterion) and AICc (Akaike information criterion) and enabled the establishment of a relatively extensive analysis of variance model recorded in Table 3: the equation for the cutting speed (1) in used factor units was the following:

(1) $$\begin{array}{l} v_{c}=-41.79133+3.42735T_{on}+0.239849T_{off}+2.5625I+0.249378U-0.0298t\\ \;\;\;\;\;+0.007583T_{on}\cdot T_{off}-0.03307T_{on}\cdot I-0.029752T_{on}\cdot U+0.001632T_{on}\cdot t\\ \;\;\;\;\;-0.015241T_{off}\cdot I-0.001173T_{off}\cdot U-0.001173T_{off}\cdot t-0.002798I\cdot U\\ \;\;\;\;\;-0.003280I\cdot t+0.000978U\cdot t-0.280886T_{on}^{2}+0.001457T_{off}^{2}\\ \;\;\;\;\;-0.063261I^{2}-0.001184U^{2}+0.000575t^{2}+0.000318T_{on}\cdot T_{off}\cdot I\\ \;\;\;\;\;+0.000027T_{on}\cdot T_{off}\cdot t+0.000021T_{on}\cdot U\cdot t+0.000066T_{off}\cdot I\cdot U\\ \;\;\;\;\;+0.000012T_{off}\cdot I\cdot t+0.001223T_{on}^{2}\cdot I-0.000257T_{on}^{2}\cdot t-0.000233T_{on}\\ \;\;\;\;\;\cdot T_{off}^{2}+0.000233T_{on}\cdot U^{2}+7.049\cdot 10^{-6}T_{off}^{2}\cdot t+0.000124T_{off}\cdot I^{2}\\ \;\;\;\;\;+0.000044I^{2}\cdot t-9.549\cdot 10^{-6}U^{2}\cdot t+0.010862T_{on}^{3}+0.000614I^{3}-1.76\\ \;\;\;\;\;\cdot 10^{-6}t^{3}\;(\frac{mm}{min}). \end{array}$$

**Figure 5:**
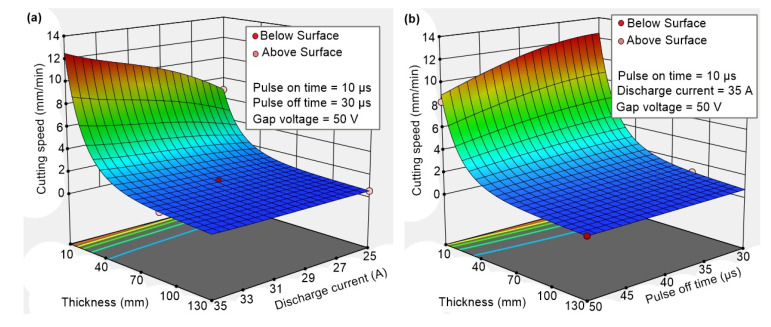
Response surfaces for (**a**) Thickness and Discharge current, (**b**) Thickness and Pulse-off time.

All significance and goodness-of-fit statistics (Table 4) are very good, including the Predicted R^2^, so the model can be used very well for predicting the cutting speed. To satisfy the lack of fit of insignificance conditions, and for the residuals in particular, the recommended transformation of the response by the natural logarithm of ln(y) was chosen. The graphs in Figure 5 show that the correct machining parameters’ settings for the 10 mm samples can lead to a cutting speed of around 12 mm/min. The setting of *T_on_* = 10 µs, *T_off_* = 30 µs, *I* = 35 A, *U* = 50 V or even *U* = 70 V, and *v* = 12 m/min will always lead to a cutting speed higher than 0.98 mm/min for samples of up to 100 mm thickness.

### 3.2. Evaluation of the Surfaces’ Topography

**Figure 6:**
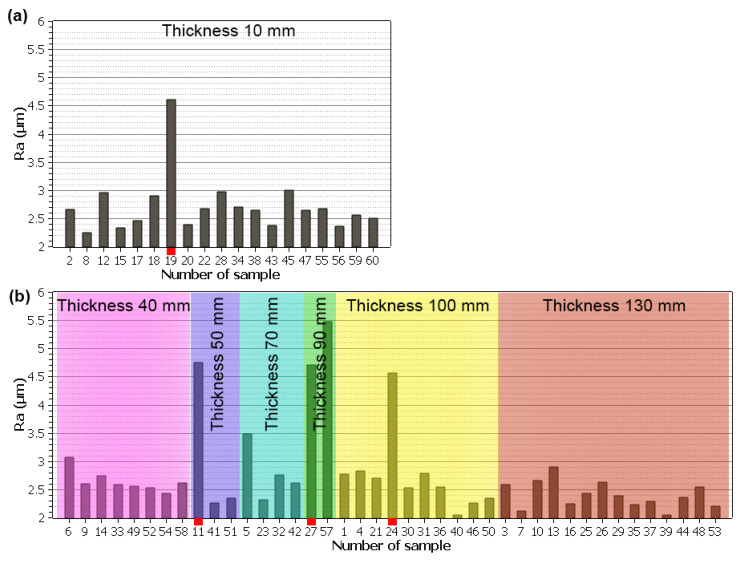
Ra values of individual samples of different thicknesses marked with a red square corresponding with the sample Ra values of from 4.5 to 5 µm, (**a**) sample thickness of 10 mm, (**b**) remaining thicknesses.

The surface roughness that characterises the surface topography was represented by the arithmetical mean deviation of profile (Ra), which is the most commonly used parameter. The Ra parameter was evaluated on the surfaces of all machined samples, always in five given places, and the arithmetic mean of these values was established. The topography parameter was evaluated using the tactile profilometer Dektak XT, corresponding with the ISO 21920-2norm. The researched values for the surface roughness, Ra, in class 33 were based on the VDI 3402Blatt4 norm: the Application of Electrical Discharge Machining. The norm requires an Ra range from 4.5 to 5 µm for individual thicknesses of the material. This is because of the required relief of machined inserts and plastic injection mould segments. This relief means that the insert or segment does not need to be etched. The graphs in Figure 6 for individual material thicknesses show that the required Ra values from 4.5 to 5 µm were reached in four cases, that is, for the thicknesses of 10, 50, 90, and 100 mm. These are marked with a red square for better clarity. It is, however, visible that the other samples’ Ra values are overall lower, as presented in the study by Sathiyaraj [6]. The required Ra values from 4.5 to 5 µm were also achieved in the study by Li [10], which makes us conclude that, for the given material, these are realistic and obtainable without much difficulty.

**Figure 7:**
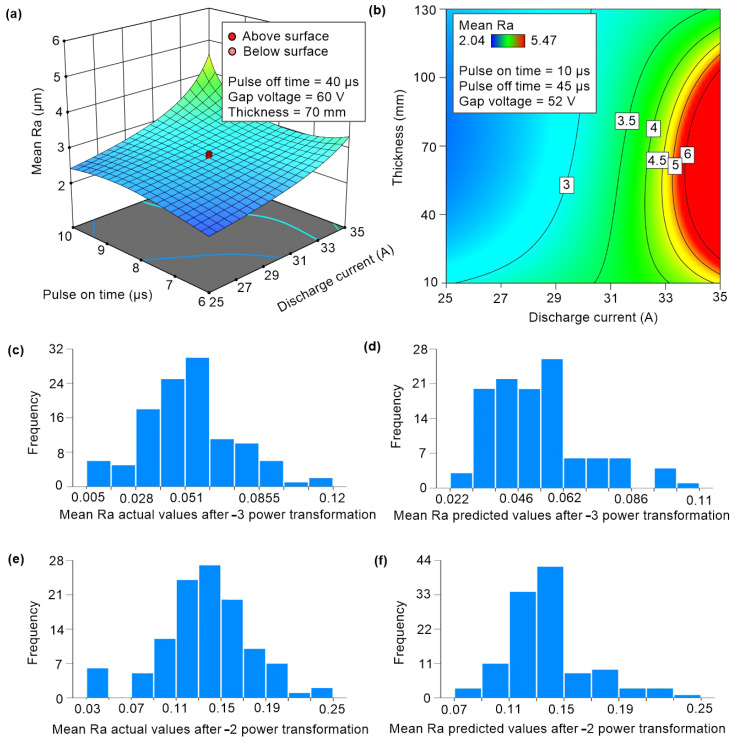
(**a**) Response surface of the factors Pulse-on time and Discharge current, (**b**) contour plot of the factors Discharge current and Thickness, (**c**,**d**) histogram of Mean Ra transformation data y^−3^, (**e**,**f**) Histogram of Mean Ra predictions from y^−2^ transformation models.

From the histogram of the measured Mean Ra values, it was found that the distribution is skewed to the right. For both Ra responses, the software from the Box-Cox graph recommended performing a power-law transformation of the response close to −2.5 to meet the assumptions of a normal distribution and homoscedasticity. When leaving the response untransformed, only models with negative Predicted R^2^ values were found, indicating that even the average of all Ra measurements alone would be a better model than the one found. Since the −2.5 power transformation is not very practical when back-transforming, this value was rounded, and the best model was found with a −3 power transformation. Several methods were used, but the best result was achieved with the backward selection with the AICc criterion, where redundant triple interactions were still manually reduced to reach better Predicted R^2^ and Adjusted R^2^ values. Moreover, the B^2^C member was included in the model, which did not appear to be statistically significant but always figured in all other models with *p*-values below 0.05. Adding this member brought an improvement to the statistics of the goodness-of-fit R^2^, Adjusted R^2^, and insignificance of the lack of fit residuals. Contrarily, the statistics of BIC, AIC, and Predicted R^2^ became slightly worse. The B^2^C member was fairly correlated with the other A^2^C, CD^2^, and CE^2^ members but did not significantly increase the multicollinearity of the model. The Variance Inflation Factor (VIF) statistic is fine, and the Pearson correlation with the other members of the model does not exceed 0.8. This is a common threshold in the field of machine learning for omitting a correlated factor from a dataset due to other factors carrying very similar information. The B^2^C term remained in the model because it made it possible to observe how the discharge current factor, which is known to influence all other factors, also affected the B^2^ quadratic term. Additionally, it was discovered that with the B^2^C term in the model, the B^3^ cubic term becomes significant. The contour plot of the model in Figure 7b shows that it is possible to achieve an average roughness, Ra, in the range of 4.5 to 5 µm, according to the norm for all samples of thickness from 10 to 100 mm. However, this is a fairly narrow yellow band from the entire experimental space that the machining parameters must meet. The greatest impact on the Mean Ra response change was seen in the following terms of the equation in descending order: CE^2^, B, B^3^, A^2^C, CD^2^, C, A, B^2^C, E, E^2^, A^2^, AC, B^2^, CE, D, D^2^, BC, and CD. The response surface in Figure 7a shows the dependence between factors A and C, which are among the most important and which will be able to achieve the required response.

During the optimisation, the software revealed that finding a suitable machine setting would be difficult as it was a high-order and high-power model at the same time. The yellow band of the contour plot (Figure 7b) shows the average values for the back-detransformed response. The distortion could be caused by this mathematical operation itself. In the back-transformation, the software applies a correction to again ‘skew’ the mean value of the distribution to the right of the median. This correction appeared to be too strong, as the histogram in Figure 7d does not resemble a Gaussian curve nor the original Mean Ra distribution. Therefore, it was decided to use the model given in Table 5 with the goodness-of-fit statistics given in Table 6 in order to optimise and predict the correct setting. The Mean Ra Equation (2) is the following:

(2) $$\begin{array}{l} {\left(Mean\;Ra\right)}^{-2}=5.613261-0.887581\cdot T_{on}+0.253616\cdot T_{off}-0.251031\cdot I\\ \;\;\;\;\;\;\;\;\;\;-0.196489\cdot U+0.011156\cdot tl+0.028487\cdot T_{on}\cdot I-0.002921\cdot T_{off}\cdot I\\ \;\;\;\;\;\;\;\;\;\;+0.006675\cdot I\cdot U-0.000358\cdot I\cdot t+0.054236\cdot {T_{on}}^{2}-0.005375\cdot {T_{off}}^{2}\\ \;\;\;\;\;\;\;\;\;\;+0.001624\cdot U^{2}-7.675428\cdot 10^{-5}\cdot t^{2}-0.001757\cdot {T_{on}}^{2}\cdot I\;\;\;\;\;+3.643677\\ \;\;\;\;\;\;\;\;\;\;\cdot 10^{-6}\cdot {T_{off}}^{2}\cdot I-5.524470\cdot 10^{-5}\cdot I\cdot U^{2}+2.503271\cdot 10^{-6}\cdot I\cdot t^{2}\\ \;\;\;\;\;\;\;\;\;\;+3.604859\cdot 10^{-6}\cdot {T_{off}}^{3}(µm) \end{array}$$

### 3.3. Optimisation

The Mean Ra response was set to a target value of 4.5 throughout the optimisation process as it is the median of the transformed response (the median and mean of a normal distribution are the same values). The average Mean Ra value for the given machining parameters ought to be higher, and it is predicted in Table 7 in the last Mean Ra column. The greatest emphasis (importance) of five was placed on optimising the Mean Ra, as shown in Table 8. Optimisation with the setting of criteria according to weights is a widely used method presented in many publications, such as Gowd [21], Reddy [22], or Kavimani [23].

Table 8 summarises the recommended combinations according to the required optimisation with the highest ‘desirability function’, which evaluates the extent to which the specified requirements have been met. The lower transformation response model does not predict satisfactory machining parameters for samples of 10 mm and 100 mm thickness. Therefore, the actual machining parameters were filled in for sample No. 19 with a 10 mm thickness and sample No. 24 with a 100 mm thickness. Trial samples were made based on these machining parameters to check the optimisation. The achieved results were in accordance with the predicted responses and differed very minorly.

### 3.4. Morphology of Machined Surfaces and Chemical Composition Analysis

**Figure 8:**
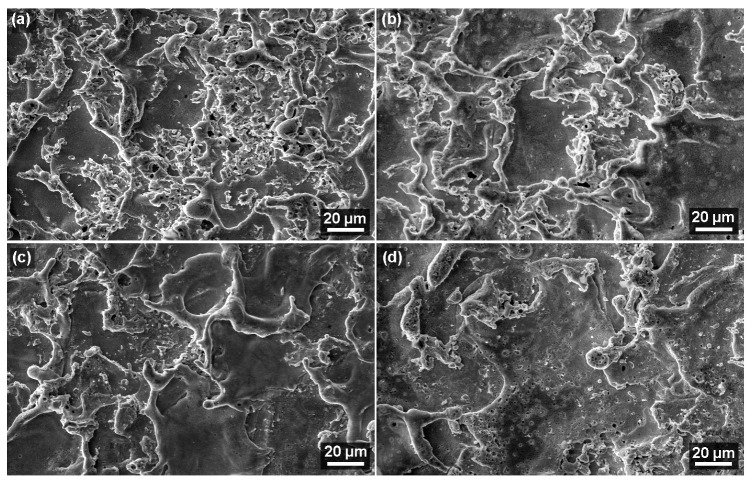
Surface morphology of samples at a magnification of 1000× (SEM/SE), (**a**) sample No. 19 with a thickness of 10 mm, (**b**) sample No. 11 with a thickness of 50 mm, (**c**) sample No. 27 with a thickness of 70 mm, (**d**) sample No. 24 with a thickness of 100 mm.

The morphology of an electro-erosion machined surface comprises a large number of randomly placed craters caused by the electric discharges. These craters can be of different sizes, as shown in the study by Tosun [24], have different shapes, as shown in the study by Esteves [25], and have different depths, as shown by Bisaria [26]. In all cases, a secondary electron detector (SE) was used for imaging at a basic magnification of 1000× and then 2500×. Figure 8 shows the surface morphology of samples No. 19, 11, 27, and 24, which were of different thicknesses that met the Ra parameter of 4.5 to 5 µm. These images allow us to see that the craters are the most rugged in sample No. 19 and the least in sample No. 24. Rugged craters are typical for steels [27], while smoother ones are typical for shape-memory alloys [28]. All surfaces in Figure 8 meet the Ra range of 4.5 to 5 µm, most likely because Ra was measured in five different places, and then the average of these values was established. Nonetheless, all the surfaces of the samples produced as part of the planned experiment show no signs of cracks, which is very positive in terms of the service life of the produced parts and their expected functionality. Electro-erosively machined surfaces often suffer from cracks, as seen in almost pure titanium [29] or aluminium alloy 6061 [30].

### 3.5. Subsurface Area Analysis

**Figure 9:**
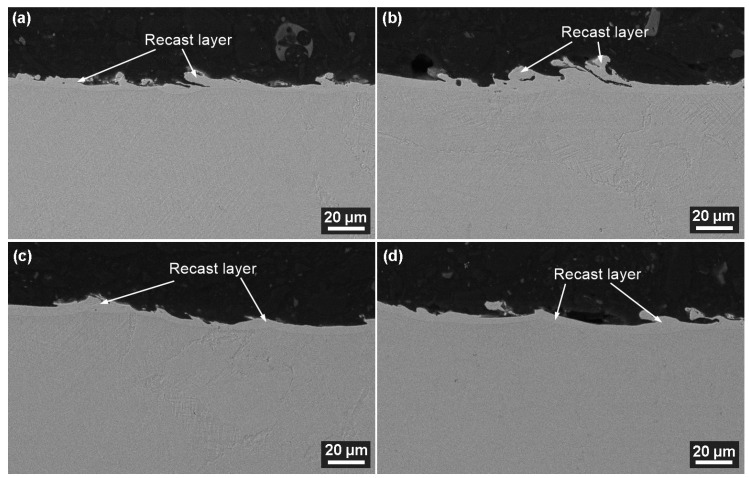
Cross-section of samples at a magnification of 1000× (SEM/SE), (**a**) sample No. 19 with a thickness of 10 mm, (**b**) sample No. 11 with a thickness of 50 mm, (**c**) sample No. 27 with a thickness of 70 mm, (**d**) sample No. 24 with a thickness of 100 mm.

Electro-erosively machined surfaces are very susceptible to the formation of subsurface cracks, which can only be uncovered when examining metallographic preparations of cross-sections of the sample. These cracks are caused by the residual charge after machining and were presented in the studies by Salvati [31] and Zhang [32]. The residual charge comes from the short-term exposure to very high temperatures of 10,000 to 20,000 °C [33], which cause the surface layer to melt and, when cooled again by the dielectric liquid, cause it to solidify. A backscattered electron detector (BSE) was used for the imaging of the cross-sections that were always studied at a magnification of 1000× first and then 2500×. Figure 9 shows the cross-sections that met the required Ra value from 4.5 to 5 µm. No defects were studied on any of the produced samples, which is a very positive fact. This means that they will have no effect on the expected service life or the proper functionality of the parts. The images show a recast layer, which is a continuous layer covering all the produced samples without exception. The recast layer thickness varies from 5 µm to 25 µm.

## 4. Conclusions

The subject of this study was the WEDM machinability of different thicknesses of Albromet W130 to the required surface roughness in a range of Ra from 4.5 to 5 µm. An extensive planned experiment of 60 rounds was carried out, leading to the following conclusions:

- As the thickness increased, the cutting speed decreased; the highest cutting speed of 11.6 mm/min was seen in sample No. 28 with the following machining parameters: *T_on_* = 10 µs, *T_off_* = 50 µs, *I* = 25 A, *U* = 70 V, and *v* = 12 m/min.
- The required Ra values from 4.5 to 5 µm were reached in four cases; that is, for the thicknesses of 10, 50, 90, and 100 mm.
- An adequate model for response behaviour, that is, for the cutting speed and surface roughness, was established, along with the corresponding equations.
- An optimisation according to the set criteria was performed, and the machining parameters for the thicknesses of 10 to 100 mm (past 10 mm) were established for the samples to meet the required surface roughness range.
- The surface and subsurface layer analysis did not discover any defects, which is very positive, as it means that the expected service life and correct functionality of the produced workpieces will not be affected.

All the above-mentioned conclusions allow us to state that the WEDM parameters were established for different thicknesses of Albromet W130 so that the Ra met the requirements without the need for subsequent etching. The main advantage is that the parameters have been clearly specified, which allows all material thicknesses to be within the required Ra value from 4.5 to 5 µm. This would not have been possible without the knowledge published in this article. Further research will focus on other thickness ranges that are used in the automotive industry.

## Figures and Tables

**Table 1:** Proposed I-optimal design of experiment for the cubic model.

No. of Sample	Type	Pulse-OnTime (µs)	Pulse-Off Time (µs)	Discharge Current (A)	Gap Voltage (V)	Wire Speed (m/min)	Thickness (mm)
1	Model	9	45	28	55	12	100
2	Model	7	30	35	50	12	10
3	Model	6	37	32	70	12	130
4	Replicate	9	45	33	65	12	100
5	Centre	8	40	30	60	12	70
6	Model	7	45	28	65	12	40
7	Lack of Fit	6	50	25	50	12	130
8	Model	6	50	25	57	12	10
9	Model	7	35	33	65	12	40
10	Model	10	37	28	70	12	130
11	Model	6	37	35	50	12	50
12	Lack of Fit	10	50	35	70	12	10
13	Lack of Fit	10	50	35	50	12	130
14	Model	9	35	33	55	12	40
15	Lack of Fit	6	50	25	70	12	10
16	Model	9	30	32	50	12	130
17	Lack of Fit	10	30	25	70	12	10
18	Model	10	37	32	70	12	10
19	Model	10	50	25	70	12	10
20	Model	7	50	25	50	12	10
21	Replicate	9	45	28	55	12	100
22	Lack of Fit	6	30	35	70	12	10
23	Centre	8	40	30	60	12	70
24	Model	9	45	33	65	12	100
25	Lack of Fit	10	30	25	50	12	130
26	Model	10	50	25	50	12	130
27	Model	6	30	35	70	12	90
28	Model	10	30	35	70	12	10
29	Lack of Fit	6	30	35	50	12	130
30	Model	7	45	33	65	12	100
31	Model	9	35	33	65	12	100
32	Centre	8	40	30	60	12	70
33	Model	9	45	28	55	12	40
34	Model	6	50	35	70	12	10
35	Model	6	50	25	63	12	130
36	Replicate	7	35	28	55	12	100
37	Lack of Fit	10	50	25	70	12	130
38	Model	10	50	25	57	12	10
39	Lack of Fit	6	30	25	70	12	130
40	Model	7	35	28	65	12	100
41	Model	6	43	25	50	12	50
42	Centre	8	40	30	60	12	70
43	Model	6	37	28	50	12	10
44	Model	6	50	35	50	12	130
45	Model	10	50	35	50	12	10
46	Model	7	35	33	55	12	100
47	Model	6	50	35	50	12	10
48	Model	9	50	35	63	12	130
49	Replicate	7	45	33	55	12	40
50	Model	7	35	28	55	12	100
51	Model	6	30	25	63	12	50
52	Model	7	45	33	55	12	40
53	Model	6	30	25	50	12	130
54	Replicate	7	45	28	65	12	40
55	Model	10	30	28	57	12	10
56	Model	6	37	28	70	12	10
57	Model	10	30	35	50	12	90
58	Model	9	35	28	65	12	40
59	Model	6	30	35	57	12	10
60	Model	10	30	25	50	12	10

**Table 2:** Investigated factors of the design of experiment.

Factor	Name	Units	Min	Max	Lower Limit	Upper Limit	Centre Point
A	Pulse-on time	µs	6.00	10.00	−1 ↔ 6.00	+1 ↔ 10.00	7.91
B	Pulse-off time	µs	30.00	50.00	−1 ↔ 30.00	+1 ↔ 50.00	40.04
C	Discharge current	A	25.00	35.00	−1 ↔ 25.00	+1 ↔ 35.00	30.02
D	Gap voltage	V	50.00	70.00	−1 ↔ 50.00	+1 ↔ 70.00	59.71
E	Thickness	mm	10.00	130.00	−1 ↔ 10.00	+1 ↔ 130.00	67.72

**Table 3:** ANOVA results for cutting speed.

Source	Sum of Squares	df	Mean Square	F-Value	*p*-Value	
Block	1.07	1	1.07			
Model	118.85	36	3.30	1563.65	<0.0001	significant
A—Pulse-on time	0.0043	1	0.0043	2.03	0.1588	
B—Pulse-off time	0.4571	1	0.4571	216.49	<0.0001	
C—Discharge current	0.0147	1	0.0147	6.97	0.0101	
D—Gap voltage	0.0122	1	0.0122	5.76	0.0188	
E—Thickness	2.12	1	2.12	1003.35	<0.0001	
AB	0.0016	1	0.0016	0.7441	0.3911	
AC	0.0031	1	0.0031	1.46	0.2307	
AD	0.0024	1	0.0024	1.12	0.2931	
AE	0.0165	1	0.0165	7.82	0.0066	
BC	0.0193	1	0.0193	9.14	0.0034	
BD	0.0105	1	0.0105	4.99	0.0285	
BE	0.0110	1	0.0110	5.23	0.0250	
CD	0.0022	1	0.0022	1.04	0.3106	
CE	0.0537	1	0.0537	25.45	<0.0001	
DE	0.0003	1	0.0003	0.1314	0.7180	
A^2^	0.0009	1	0.0009	0.4184	0.5197	
B^2^	0.0016	1	0.0016	0.7518	0.3886	
C^2^	7.400 × 10^−6^	1	7.400 × 10^−6^	0.0035	0.9529	
D^2^	0.0000	1	0.0000	0.0077	0.9302	
E^2^	9.18	1	9.18	4346.13	<0.0001	
ABC	0.0231	1	0.0231	10.94	0.0014	
ABE	0.0218	1	0.0218	10.34	0.0019	
ADE	0.0117	1	0.0117	5.54	0.0212	
BCD	0.0206	1	0.0206	9.75	0.0025	
BCE	0.0239	1	0.0239	11.31	0.0012	
A^2^C	0.0080	1	0.0080	3.77	0.0558	
A^2^E	0.0337	1	0.0337	15.96	0.0001	
AB^2^	0.0229	1	0.0229	10.85	0.0015	
AD^2^	0.0168	1	0.0168	7.95	0.0061	
B^2^E	0.0188	1	0.0188	8.89	0.0038	
BC^2^	0.0109	1	0.0109	5.15	0.0260	
C^2^E	0.0264	1	0.0264	12.48	0.0007	
D^2^E	0.0329	1	0.0329	15.59	0.0002	
A^3^	0.0175	1	0.0175	8.30	0.0052	
C^3^	0.0145	1	0.0145	6.86	0.0106	
E^3^	0.3247	1	0.3247	153.79	<0.0001	
Residual	0.1605	76	0.0021			
Lack of Fit	0.1214	51	0.0024	1.53	0.1264	not significant
Pure Error	0.0390	25	0.0016			
Cor Total	120.08	113				

**Table 4:** Goodness-of-fit statistics for cutting speed.

Std. Dev.	0.046	R^2^	0.9987
Mean	0.37	Adjusted R^2^	0.9980
C.V. %	12.43	Predicted R^2^	0.9966
		Adeq Precision	128.4728

**Table 5:** ANOVA results for Mean Ra^−2^ (Reduced Cubic Model Transform) response: Power, Lambda: −2, Constant: 0).

Source	Sum of Squares	df	Mean Square	F-Value	*p*-Value	
Block	0.0196	1	0.0196			
Model	0.0793	18	0.0044	5.51	<0.0001	significant
A—Pulse-on time	0.0187	1	0.0187	23.36	<0.0001	
B—Pulse-off time	0.0036	1	0.0036	4.54	0.0357	
C—Discharge current	0.0060	1	0.0060	7.50	0.0074	
D—Gap voltage	0.0003	1	0.0003	0.3875	0.5351	
E—Thickness	0.0065	1	0.0065	8.10	0.0054	
AC	0.0005	1	0.0005	0.6335	0.4281	
BC	3.157 × 10^−6^	1	3.157 × 10^−6^	0.0039	0.9500	
CD	0.0002	1	0.0002	0.2178	0.6418	
CE	0.0002	1	0.0002	0.3015	0.5843	
A^2^	0.0006	1	0.0006	0.7292	0.3953	
B^2^	0.0004	1	0.0004	0.5283	0.4691	
D^2^	0.0002	1	0.0002	0.2321	0.6311	
E^2^	0.0008	1	0.0008	0.9395	0.3349	
A^2^C	0.0110	1	0.0110	13.71	0.0004	
B^2^C	0.0036	1	0.0036	4.44	0.0377	
CD^2^	0.0077	1	0.0077	9.58	0.0026	
CE^2^	0.0120	1	0.0120	15.05	0.0002	
B^3^	0.0034	1	0.0034	4.28	0.0414	
Residual	0.0752	94	0.0008			
Lack of Fit	0.0619	69	0.0009	1.68	0.0740	not significant
Pure Error	0.0133	25	0.0005			
Cor Total	0.1741	113				

**Table 6:** Goodness-of-fit statistics for Mean Ra^−2^ response.

Std. Dev.	0.0283	R^2^	0.5133
Mean	0.1380	Adjusted R^2^	0.4201
C.V. %	20.50	Predicted R^2^	0.2158
		Adeq Precision	14.2098

**Table 7:** Goals, range, weights, and importance of the optimised responses and factors.

Name	Goal	Lower Limit	Upper Limit	Lower Weight	Upper Weight	Importance
Pulse-on time	Is in range	6	10	1	1	3
Pulse-off time	Is in range	30	50	1	1	3
Discharge current	Is in range	25	35	1	1	3
Gap voltage	Is in range	50	70	1	1	3
Thickness	Is target = X	10	100	1	1	3
Cutting speed	None	0.38	11.6	1	1	3
Mean Ra (median)	Is target = 4.5	4.5	5	10	1	5

**Table 8:** Optimal machining parameters for different sample thicknesses.

	Pulse-On Time (µs)	Pulse-Off Time (µs)	Discharge Current (A)	Gap Voltage (V)	Wire Speed (m/min)	Thickness (mm)	Cutting Speed (mm/min)	Mean Ra (µm)
1	10	50	25	70	12	10	5.5	4.6
2	10	42.4	34.9	50	12	20	7.2	5.05
3	9.8	41.8	35	50	12	30	3.3	5.05
4	9.6	45.4	35	50.5	12	40	2.0	5.05
5	9.8	43.5	34.1	50	12	50	1.6	5.05
6	9.8	47	34.4	50	12	60	1.2	5.05
7	9.8	45.6	34.4	51.1	12	70	1.1	5.08
8	9.9	44.7	34.9	52.9	12	80	0.9	5.05
9	10	45.3	34.8	51.7	12	90	0.8	5.05
10	9	45	33	65	12	100	0.7	4.56

## Data Availability

The raw data supporting the conclusions of this article will be made available by the authors on request.

## References

[1] Ho K.H., Newman S.T., Rahimifard S., Allen R.D. (2004). State of the art in wire electrical discharge machining (WEDM). Int. J. Mach. Tools Manuf..

[2] Abbas N.M., Solomon D.G., Bahari M.F. (2007). A review on current research trends in electrical discharge machining (EDM). Int. J. Mach. Tools Manuf..

[3] Ukey K., Sahu A.R., Gajghate S.S., Behera A.K., Limbadri C., Majumder H. (2023). Wire electrical discharge machining (WEDM) review on current optimization research trends. Mater. Today Proc..

[4] Kumar N., Kumari S., Abhishek K., Nandi G., Ghosh N. (2022). Study on various parameters of WEDM using different optimization techniques: A review. Mater. Today Proc..

[5] Davis J.R. (2001). Copper and Copper Alloys.

[6] Sathiyaraj S., Venkatesan S., Ashokkumar S. (2020). Wire electrical discharge machining (WEDM) analysis into MRR and SR on copper alloy. Mater. Today Proc..

[7] Thankachan T., Soorya Prakash K., Loganathan M. (2018). WEDM process parameter optimization of FSPed copper-BN composites. Mater. Manuf. Process..

[8] Katiyar J.K., Sharma A.K., Pandey B. (2018). Synthesis of iron-copper alloy using electrical discharge machining. Mater. Manuf. Process..

[9] Li W., Li Z. (2022). Experimental research on WEDM of copper tungsten alloy based on orthogonal test. J. Phys. Conf. Ser..

[10] Li W.M. (2020). Experimental study on cutting speed and surface roughness of pure copper in WEDM. J. Phys. Conf. Ser..

[11] Ahmed N., Mughal M.P., Shoaib W., Farhan Raza S., Alahmari A.M. (2020). WEDM of copper for the fabrication of large surface-area micro-channels: A prerequisite for the high heat-transfer rate. Micromachines.

[12] Ali M.A., Samsul M., Hussein NI S., Rizal M., Izamshah R., Hadzley M., Kasim M.S., Sulaiman M.A., Sivarao S. (2013). The effect of EDM die-sinking parameters on material removal rate of beryllium copper using full factorial method. Middle-East J. Sci. Res..

[13] Bhuiyan M., Shihab B. (2016). Development of copper based miniature electrostatic actuator using WEDM with low actuation voltage. Microsyst. Technol..

[14] Mouralova K., Benes L., Zahradnicek R., Bednar J., Hrabec P., Prokes T., Matousek R., Fiala Z. (2018). Quality of surface and subsurface layers after WEDM aluminum alloy 7475-T7351 including analysis of TEM lamella. Int. J. Adv. Manuf. Technol..

[15] Mouralova K., Benes L., Zahradnicek R., Bednar J., Zadera A., Fries J., Kana V. (2020). WEDM Used for machining high entropy alloys. Materials.

[16] Mouralova K., Benes L., Bednar J., Zahradnicek R., Prokes T., Fiala Z., Fries J. (2020). Precision machining of nimonic C 263 super alloyusing WEDM. Coatings.

[17] Mouralova K., Bednar J., Benes L., Prokes T., Zahradnicek R., Fries J. (2022). Mathematical models for machining optimization of Ampcoloy 35 with different thicknesses using WEDM to improve the surface properties of mold parts. Materials.

[18] Liao Y.S., Chuang T.J., Yu Y.P. (2013). On-line workpiece height estimation and its application in servo feed control of WEDM process. Procedia CIRP.

[19] Manoj I.V., Joy R., Narendranath S. (2020). Investigation on the effect of variation in cutting speeds and angle of cut during slant type taper cutting in WEDM of Hastelloy X. Arab. J. Sci. Eng..

[20] Singh B., Misra J.P. (2018). Empirical modeling of average cutting speed during WEDM of gas turbine alloy. MATEC Web Conf..

[21] Gowd G.H., Reddy M.G., Sreenivasulu B., Ravuri M. (2014). Multi objective optimization of process parameters in WEDM during machining of SS304. Procedia Mater. Sci..

[22] Reddy B.S., Rao A.K., Janardhana G.R. (2021). Multi-objective optimization of surface roughness, recast layer thickness and surface crack density in WEDM of Al2124/SiCp using desirability approach. Mater. Today Proc..

[23] Kavimani V., Prakash K.S., Thankachan T. (2019). Multi-objective optimization in WEDM process of graphene–SiC-magnesium composite through hybrid techniques. Measurement.

[24] Tosun N., Pihtili H. (2003). The effect of cutting parameters on wire crater sizes in wire EDM. Int. J. Adv. Manuf. Technol..

[25] Esteves P.M., Wiessner M., Costa J.V., Sikora M., Wegener K. (2021). WEDM single crater asymmetry. Int. J. Adv. Manuf. Technol..

[26] Bisaria H., Shandilya P. (2019). Study on crater depth during material removal in WEDC of Ni-rich nickel–titanium shape memory alloy. J. Braz. Soc. Mech. Sci. Eng..

[27] Tahir W., Jahanzaib M., Ahmad W., Hussain S. (2019). Surface morphology evaluation of hardened HSLA steel using cryogenic-treated brass wire in WEDM process. Int. J. Adv. Manuf. Technol..

[28] Chaudhari R., Vora J.J., Patel V., Lacalle LL D., Parikh D.M. (2020). Effect of WEDM process parameters on surface morphology of nitinol shape memory alloy. Materials.

[29] Kumar A., Kumar V., Kumar J. (2016). Surface crack density and recast layer thickness analysis in WEDM process through response surface methodology. Mach. Sci. Technol..

[30] Rouniyar A.K., Shandilya P. (2022). Study of surface crack density and microhardness of Aluminium 6061 alloy machined by EDM with mixed powder and assisted magnetic field. J. Micromanufacturing.

[31] Salvati E., Korsunsky A.M. (2020). Micro-scale measurement & FEM modelling of residual stresses in AA6082-T6 Al alloy generated by wire EDM cutting. J. Mater. Process. Technol..

[32] Zhang G., Li W., Zhang Y., Huang Y., Zhang Z., Chen Z. (2020). Analysis and reduction of process energy consumption and thermal deformation in a micro-structure wire electrode electric discharge machining thin-wall component. J. Clean. Prod..

[33] Guo C., Wu Z., Wang X., Zhang J. (2021). Comparison in performance by emulsion and SiC nanofluids HS-WEDM multi-cutting process. Int. J. Adv. Manuf. Technol..

